# Immune checkpoint inhibition in bone metastases: lesion response heterogeneity, the osteoimmune niche, and microenvironment-directed therapy

**DOI:** 10.3389/fimmu.2026.1959101

**Published:** 2026-09-17

**Authors:** Min Dong, Guili Zheng, Fangyuan Yu, Jiaqi Yin, Chen Zhong

**Affiliations:** 1 Department of Medical Oncology, The 960th Hospital of the People's Liberation Army (PLA) Joint Logistic Support Force, Jinan, Shandong, China; 2 Jinzhou Medical University, Jinzhou, China

**Keywords:** bone metastases, immune checkpoint inhibitors, lesion-level response heterogeneity, osteoimmune niche, response assessment

## Abstract

Bone metastases are common in advanced solid tumors and are clinically important not only because they cause skeletal-related events, including bone pain, pathological fractures, and spinal cord compression, but also because they may influence responses to immune checkpoint inhibitors (ICIs). Clinical evidence has consistently associated bone metastases with poorer survival after ICI treatment. Although some studies suggest that the relative benefit of ICIs may be reduced in patients with bone metastases, most available evidence is retrospective and lacks adequate treatment-interaction testing. This apparent reduction in benefit therefore cannot be clearly distinguished from adverse baseline prognosis, greater disease burden, or confounding related to concomitant treatments. Lesion-level studies further suggest that bone and nonbone lesions may follow discordant response trajectories. Assessment of bone-lesion response is complicated by the limited measurability of bone-only lesions under RECIST 1.1 and by treatment-related sclerosis, bone flare, and mixed responses. Mechanistically, bone metastases may establish a site-specific osteoimmune niche in which abnormal bone remodeling, vascular and stromal reorganization, myeloid immunosuppression, and impaired T-cell infiltration and effector function jointly constrain local antitumor immunity. RANKL inhibition and bone-directed radiotherapy are clinically available and may have immunomodulatory effects, but current evidence does not establish that either approach consistently enhances the antitumor activity of ICIs beyond skeletal protection or local control. Most other microenvironment-directed strategies remain at the preclinical or early translational stage. Future studies should distinguish bone-lesion antitumor response from systemic disease control and skeletal clinical outcomes. Prespecified treatment-interaction analyses, longitudinal multimodal imaging, paired sampling of bone and nonbone lesions, and mechanism-embedded clinical trials will be required to determine whether specific osteoimmune niche states can guide patient selection and combination therapy.

## 1 Introduction

Immune checkpoint inhibitors (ICIs) have reshaped the treatment landscape of multiple advanced solid tumors, yet bone metastases remain a major blind spot in response assessment. Patients with bone metastases often experience poorer survival and disease-control outcomes after ICI treatment (1–5), but the meaning of this association remains uncertain. It may reflect greater overall tumor burden, poorer performance status, and a higher frequency of skeletal complications, or it may suggest that bone lesions are partly protected by an organ-specific microenvironment. At the same time, bone-only lesions often lack a measurable soft-tissue component, while treatment-related sclerosis, remineralization, and bone flare may be misclassified as tumor progression (6–10). Poor outcomes in patients with bone metastases may therefore reflect biological differences, differences in patient characteristics, limitations in response assessment, or a combination of these factors.

The available evidence should be interpreted at three distinct levels. First, the association between bone metastases and poorer overall survival (OS) or progression-free survival (PFS) in ICI-treated cohorts primarily reflects prognostic significance. Second, determining whether bone metastases reduce the benefit of ICIs relative to chemotherapy or other treatments requires comparison of relative treatment effects across groups and formal treatment-interaction testing. Subgroup analyses from randomized trials suggest that patients with bone metastases still benefit from ICIs, but they do not establish that the magnitude of benefit is truly lower than that observed in patients without bone metastases (11). Third, patient-level outcomes such as OS, PFS, and objective response rate cannot directly characterize the response of bone lesions themselves. Some lesion-level studies have reported differences in response duration and progression patterns between bone and nonbone metastases (7, 8, 12–14), but these findings are also influenced by lesion measurability, imaging interpretation, and selective follow-up. Adverse prognosis, treatment-effect modification by bone metastases, and reduced lesion-level responsiveness within bone should therefore be treated as separate questions.

Previous reviews have generally described the bone metastatic microenvironment through immune-cell populations, cytokines, or signaling pathways, or have summarized ICIs, denosumab, radiotherapy, and other combination strategies separately (15–23). These studies have provided important mechanistic information, but the evidentiary boundaries between clinical associations, lesion-level responses, and therapeutic interventions remain unclear. This review does not assume that bone metastases constitute an established site of ICI resistance. Instead, it follows a hierarchy of clinical reasoning. We first assess whether bone metastases are best understood as a prognostic marker, a potential modifier of treatment effect, or a site of relative low sensitivity in only a subset of patients. We then examine whether current imaging methods can reliably detect such differences and whether abnormal bone remodeling, myeloid immunosuppression, vascular and stromal remodeling, and impaired T-cell function provide cancer- and lesion-specific mechanistic explanations. Finally, we evaluate whether RANKL inhibition, bone-directed radiotherapy, and other microenvironment-directed interventions produce antitumor benefits beyond skeletal protection or local control, and define the clinical and biological evidence required to establish the therapeutic relevance of the osteoimmune niche.

## 2 Clinical evidence: adverse prognosis, uncertain treatment interaction, and lesion-level heterogeneity

The available clinical evidence supports a relatively clear but limited conclusion: bone metastases are an adverse risk marker in ICI-treated populations, but they have not been established as an ICI-specific predictive factor (**Table 1**). This distinction is critical. Poor survival after ICI treatment may reflect more extensive disease, reduced bone marrow and functional reserve, and a greater burden of skeletal complications. It may also reflect reduced local immune responsiveness within bone lesions. Distinguishing these explanations requires treatment comparisons with an appropriate control group and formal interaction testing. Lesion-level studies further suggest that bone and nonbone lesions may follow discordant response trajectories, although this signal remains constrained by lesion selection and imaging methods.

**Table 1:** Clinical evidence on bone metastases and outcomes during immune checkpoint inhibition.

Cancer type	Evidence setting	BM comparison	Endpoint/interaction	Key finding/interpretation	Study
NSCLC	RCT meta-analysis; ICI vs control	Treatment effect by BM status	OS, PFS/NR	ICI benefit persisted in BM+, but appeared numerically attenuated; formal treatment interaction was not reported	(24)
NSCLC	Meta-analysis of ICI cohorts	BM+ vs BM−	OS, PFS/No	BM was associated with poorer survival — prognostic association	(2)
RCC	Phase III subgroup; nivolumab vs everolimus	Treatment effect by BM status	OS/NR	Nivolumab benefit was maintained in BM+; formal treatment interaction was not reported	(25)
RCC	Multicenter nivolumab cohort	BM+ vs BM−	OS/No	BM independently predicted shorter survival — prognostic association	(26)
Urothelial carcinoma	Phase III *post hoc* analysis	BM+ vs BM− across arms	OS, PFS/No	BM was associated with worse outcomes across arms — prognostic association	(27)
Urothelial carcinoma	Organ-specific pembrolizumab cohort	Bone vs other sites	ORR/N/A	Bone lesions showed markedly lower response — lesion-level heterogeneity	(28)
Melanoma	Lesion-level ICI cohort	Bone vs other sites	Response, PFS/N/A	Response differed substantially by metastatic site — lesion-level heterogeneity	(29)
NSCLC/RCC/melanoma	Long-term nivolumab cohort	BM+ vs BM−	5-y OS/No	BM independently predicted lower long-term survival — prognostic association	(30)
Multiple cancers	Bone-imaging ICI cohort	Bone vs overall response	Bone response/N/A	Bone response could diverge from RECIST — lesion-level heterogeneity	(8)
Multiple cancers	Systematic review	Bone response across cancers	Bone response/N/A	Response varied by tumor type and assessment method — lesion-level heterogeneity	(13)

### 2.1 Bone metastases are a prognostic marker in ICI-treated populations

Systematic reviews, meta-analyses, and real-world cohorts have consistently shown that patients with NSCLC and bone metastases generally experience poorer OS after ICI treatment (1–5, 11, 31, 32). In some studies, bone metastases remained associated with poorer outcomes after adjustment for ECOG performance status, treatment line, liver or brain metastases, and the number of involved metastatic organs (1, 3–5). These findings indicate that bone metastases identify a high-risk subgroup within ICI-treated populations.

The consistency of the association with OS should not, however, be interpreted as evidence of biological resistance to ICIs. Compared with patients without bone metastases, those with bone involvement often have a greater overall tumor burden, a higher prevalence of multiorgan disease, poorer performance status, and more skeletal complications (1, 4, 31, 33–44). Bone marrow involvement, pathological fractures, spinal cord compression, and the need for radiotherapy or surgery may further compromise hematopoietic reserve, disrupt treatment continuity, and reduce opportunities for subsequent therapy. An additional limitation is that most clinical studies treat bone metastasis as a binary BM+/BM− exposure despite substantial biological heterogeneity. Osteolytic, osteoblastic, and mixed lesions, as well as direct bone marrow involvement, may differ in remodeling activity, hematopoietic consequences, stromal composition, and immune context. Yet most studies have not adequately documented these features, together with the number of bone lesions, skeletal-related events, or concomitant bone-targeted therapy. Consequently, the BM+ category in current clinical studies often combines biologically distinct skeletal states, leaving important heterogeneity unresolved even after multivariable adjustment.

Associations with OS have generally been more consistent than those with PFS, objective response rate, or disease-control rate (2, 11, 13, 45). This discordance across endpoints should, however, be interpreted cautiously. PFS remains a patient-level endpoint and may be driven by progression at nonbone sites, whereas objective response rate may systematically underrepresent bone-lesion response because many bone lesions are nonmeasurable under RECIST 1.1. Inconsistency in PFS or objective response rate therefore does not exclude the possibility of reduced responsiveness within bone lesions. Rather, it highlights the limited ability of patient-level endpoints to distinguish local bone-lesion sensitivity from overall disease burden and progression at other metastatic sites. The most defensible interpretation of current observational evidence is that bone metastases identify an ICI-treated population with a higher baseline risk. Patient-level survival data cannot determine how much of that risk is attributable to reduced immune responsiveness within bone lesions themselves.

### 2.2 Evidence for treatment-effect modification remains insufficient

Determining whether bone metastases truly reduce the relative efficacy of ICIs requires more than comparing survival between patients with and without bone metastases within ICI-treated cohorts. The relative effects of ICIs versus an appropriate comparator must be evaluated across bone-metastasis subgroups. Pooled subgroup findings from randomized trials indicate that patients with NSCLC and bone metastases still derive a survival benefit from ICIs or ICI-based combinations compared with chemotherapy (11). These findings argue against the view that bone metastases render ICIs ineffective and indicate that bone involvement alone should not be used to exclude patients from immunotherapy.

In some analyses, treatment-effect estimates were numerically weaker in patients with bone metastases than in those without bone involvement, but numerical differences alone do not establish treatment-effect heterogeneity (11). Most trials did not use bone-metastasis status as a prespecified stratification factor, bone-metastasis subgroups were often small, and formal interaction tests between treatment and bone-metastasis status were rarely reported. Statistical significance in one subgroup but not another likewise does not establish a difference in treatment effect between the subgroups. Existing pooled analyses also often combine ICI monotherapy, chemotherapy plus ICIs, different treatment lines, and populations selected using different PD-L1 criteria. The resulting average effect may therefore reflect both differences in treatment mechanism and differences in patient selection, limiting its clinical interpretation. Randomized evidence currently supports the conclusion that patients with bone metastases retain a relative benefit from ICIs. It does not establish that the magnitude of this benefit is lower than that in patients without bone metastases. The proposed attenuation of ICI benefit associated with bone metastases remains a hypothesis that requires validation through prespecified interaction testing.

### 2.3 Does treatment context explain the heterogeneity?

Differences in treatment regimen may contribute to the inconsistency of current findings. Some cohorts suggest that the adverse association of bone metastases is more pronounced with ICI monotherapy and less apparent with chemotherapy plus ICIs or other combination regimens (5, 20, 46, 47). In studies of ICI monotherapy, patients with bone metastases have often shown shorter survival or a lower probability of durable disease control (4, 5, 32, 34, 35). Much of this evidence comes from later-line nivolumab cohorts or from patients with high PD-L1 expression treated with first-line pembrolizumab monotherapy (4, 6, 34, 35). Even among patients with high PD-L1 expression, outcomes may differ according to metastatic site, suggesting that PD-L1 expression alone does not account for all organ-specific heterogeneity in treatment response.

Some studies of chemotherapy plus ICIs have not observed an adverse association of similar magnitude for bone metastases (5, 46). One possible explanation is that chemotherapy partly compensates for reduced responsiveness within bone lesions through rapid tumor reduction, antigen release, or changes in myeloid-cell populations. Current data do not support this causal interpretation. Patients receiving monotherapy and combination treatment differ systematically in PD-L1 expression, age, performance status, disease burden, and treatment indications. Cross-cohort differences therefore cannot be taken as evidence that chemotherapy reverses local immune suppression within bone.

Signals of improved bone-lesion control or survival have also been reported with ICIs combined with antiangiogenic therapy (47, 48). Antiangiogenic agents, however, have independent systemic antitumor effects, making it difficult to separate potential vascular normalization from overall tumor reduction. No paired lesion-level or longitudinal biological evidence currently shows that these combinations specifically improve the immune environment within bone. These findings suggest that treatment context may influence how bone-metastasis-associated risk is expressed, but they do not identify a regimen that clearly alters the biology of bone lesions. Distinguishing a compensatory effect of combination therapy from differences in patient selection will require direct comparisons in clinically comparable populations treated within the same therapeutic context.

### 2.4 Lesion-level evidence: relative low sensitivity or measurement artifact?

Patient-level OS and PFS cannot identify which organ or lesion drives treatment failure. Within the same patient, nonbone lesions may shrink while bone lesions remain active, or bone lesions may remain stable despite progression elsewhere. Lesion-level analyses are therefore more directly relevant to the question of whether bone represents a site of relative low sensitivity.

Discordant responses among metastatic lesions within the same patient after PD-1 blockade have been described in several studies (14, 49). In NSCLC, some studies have reported lower objective response rates, shallower responses, or shorter durations of control in bone lesions than in intrapulmonary or other nonbone lesions (6, 12, 14, 50). At the same time, ICIs can induce shrinkage of soft-tissue components, remineralization, sclerosis, or prolonged stability in some bone metastases (7, 8, 12, 13, 51). Current evidence therefore does not support the view that bone is an inherently nonresponsive site. Rather, it suggests that some bone lesions may have a lower probability of response or a shorter duration of control.

Lesion-level evidence is also susceptible to selection bias. Bone lesions included in serial quantitative analyses are often larger, easier to identify, or associated with a measurable soft-tissue component. Purely osteoblastic metastases, diffuse bone marrow involvement, and patients with early progression are frequently underrepresented. Studies also differ in how they classify sclerosis, remineralization, newly detected abnormalities on bone imaging, and previously irradiated lesions. Treatment discontinuation and cessation of follow-up after nonbone progression may further truncate observation of subsequent bone-lesion responses. Comparisons of organ-specific responses across different patients may also be confounded by differences in patient characteristics. Within-patient paired analyses of bone and nonbone lesions better control for systemic treatment exposure and host factors, but they cannot fully account for differences in lesion size, prior local treatment, imaging modality, or local drug exposure. Discordant lesion responses should therefore be viewed as a clinical signal that requires validation rather than as established evidence of bone-specific resistance. Further interpretation depends first on whether bone-lesion responses can be identified consistently and reproducibly.

## 3 Measurement bias as a potential competing explanation for apparent bone resistance

Reduced responsiveness within bone observed in lesion-level studies does not necessarily arise entirely from organ-specific treatment resistance. Imaging changes in bone metastases reflect several overlapping processes, including tumor burden, bone destruction, bone marrow infiltration, inflammation, and reparative remodeling. Current response criteria cannot reliably distinguish among these processes. Bone-only lesions are often excluded from quantitative assessment, while post-treatment sclerosis or metabolic changes may be classified as response, stable disease, or progression depending on the timing and method of evaluation. Existing assessment approaches may therefore not only miss genuine bone responses but also create an appearance of reduced bone-lesion sensitivity through lesion selection, misclassification, and nonrandom follow-up. Measurement bias should therefore be considered an important potential competing explanation for apparent bone-specific ICI resistance rather than merely a technical limitation of imaging.

### 3.1 RECIST creates structural missingness and time-dependent misclassification

RECIST 1.1 primarily evaluates response according to changes in lesion diameter. Osteolytic or mixed lesions with a measurable soft-tissue component may be designated as target lesions, whereas most purely osteoblastic metastases and bone lesions without a clearly measurable soft-tissue component can only be assessed as nontarget lesions (10). This missingness is not random. Bone lesions eligible for quantitative analysis are generally larger, easier to identify, and structurally different from diffuse bone marrow infiltration or purely osteoblastic disease. Objective response rates calculated from target lesions therefore cannot be generalized to all bone metastases, and studies of bone-lesion response may be selective at both the patient and lesion levels from the outset.

A further limitation of RECIST is that its measurement construct does not fully capture the main biological changes that occur in bone lesions after treatment. Remineralization of osteolytic areas, peripheral sclerosis, and increased bone density may indicate repair without measurable reduction in lesion diameter (7–9). Conversely, sclerosis should not automatically be interpreted as tumor response because bone-targeted therapy, radiotherapy, and chronic bone remodeling can also alter lesion structure. Without information on soft-tissue components, metabolic activity, the clinical course, and subsequent imaging, increased density on a single CT examination cannot reliably distinguish antitumor response from nonspecific bone formation. Timing adds another layer of uncertainty. Increased tracer uptake on bone scintigraphy or newly visible sclerotic lesions early during treatment may reflect healing of pre-existing lesions that have become more conspicuous, a phenomenon known as bone flare (52). Similar findings, however, may also represent true new bone metastases. In the absence of high-quality baseline imaging, a prespecified confirmation window, or follow-up examinations, the same imaging change may be classified as progression, stable disease, or response across different studies.

Immunotherapy does not resolve these limitations and may further complicate time-dependent interpretation. Importantly, iRECIST retains the RECIST 1.1 rules for lesion measurability, including the ≥10 mm soft-tissue-component threshold for measurable bone lesions, and therefore does not make bone-only lesions without a measurable soft-tissue component quantitatively assessable (10, 53). Instead, its relevant modification concerns the temporal confirmation of progression: initial RECIST-defined progression is classified as immune unconfirmed progressive disease (iUPD) and, if further progression is demonstrated on subsequent assessment, as immune confirmed progressive disease (iCPD) (53). Thus, iRECIST may reduce premature classification of atypical immune-related response patterns as definitive progression, but it addresses progression confirmation rather than bone-lesion measurability; reparative sclerosis, bone flare, and discordant lesion responses therefore remain potential sources of misclassification (8, 14, 49).

### 3.2 No alternative criterion has been validated for ICI-treated bone metastases

Supplementary imaging methods and bone-specific response criteria have attempted to address aspects not captured by RECIST. No current approach, however, simultaneously integrates bone structure, bone marrow infiltration, and tumor metabolism and has been adequately validated in the setting of ICI-treated bone metastases. CT primarily depicts bone destruction, sclerosis, and associated soft-tissue components. MRI is more sensitive for bone marrow infiltration and involvement of neural structures. Bone scintigraphy reflects osteoblastic activity, whereas PET provides information on glucose metabolism or uptake of specific molecular tracers (9, 52). These modalities do not measure the same biological process in different ways; rather, they capture distinct components of the lesion. Increased sclerosis on CT may occur alongside reduced metabolic activity on PET, while increased uptake on bone scintigraphy may reflect repair rather than tumor growth. Discordance among imaging modalities does not necessarily indicate that one method is incorrect. It may instead reflect temporal dissociation among structural change, metabolic activity, and bone remodeling.

The MD Anderson (MDA) bone response criteria were developed to capture structural and imaging changes in bone metastases that are not adequately represented by changes in lesion diameter alone. They combine qualitative and quantitative assessments across radiography, CT, MRI, and skeletal scintigraphy. Complete response (CR) includes complete sclerotic fill-in of lytic lesions or normalization of bone density on radiography or CT, normalization of signal intensity on MRI, or normalization of tracer uptake on skeletal scintigraphy. Partial response (PR) includes the development of a sclerotic rim or partial sclerotic fill-in of lytic lesions and, for measurable lesions, a ≥50% decrease in the sum of perpendicular bidimensional measurements. The criteria also explicitly recognize osteoblastic flare, in which newly visible sclerotic lesions or sclerotic rims may reflect healing rather than progression when accompanied by other signs of response and no evidence of progressive bone disease (9, 54).

In a small cohort of patients with breast cancer and bone-only metastases, the MDA criteria showed better discrimination of clinical outcomes than conventional WHO criteria (9). More recently, a retrospective single-center study of 222 patients with advanced NSCLC receiving single-agent immunotherapy found that MDA-defined bone response was associated with clinical outcomes, providing ICI-specific evidence for the potential clinical relevance of bone-response assessment (55). However, this association does not establish the MDA criteria as a validated ICI-specific response standard, particularly given the retrospective design and absence of independent prospective validation.

Importantly, the MDA criteria are not exclusively qualitative: they contain numerical thresholds for changes in measurable lesions, but several components, including sclerotic fill-in and changes in ill-defined lesions, remain qualitative or semiquantitative. Subsequent correspondence proposed more objective quantitative approaches, including CT-based density measurements, whereas the original authors acknowledged that additional quantitative measurements might improve accuracy but emphasized that exclusively quantitative criteria would render many bone metastases unmeasurable (56, 57). Interpretation may therefore remain influenced by imaging parameters, prior radiotherapy, bone-targeted therapy, lesion phenotype, and reader experience. ICI studies applying the MDA criteria have reported heterogeneous bone-response patterns (7, 8, 13, 55). Such differences cannot be attributed to treatment efficacy alone and may also reflect variation in cancer type, baseline bone burden, imaging schedule, prior treatment, and lesion-selection rules. Use of the same named criteria does not ensure that different studies are measuring the same biological event.

PERCIST and other metabolic response approaches may detect reduced metabolic activity before changes in lesion diameter, particularly in lesions without a measurable soft-tissue component. Tracer uptake, however, can also be altered by inflammation, immune-cell infiltration, bone repair, and hematopoietic stimulation. Metabolic changes during ICI treatment therefore cannot be assumed to represent a proportional reduction in tumor-cell burden. Bone scintigraphy is useful for characterizing the distribution of skeletal disease but cannot consistently distinguish active tumor from osteoblastic repair.

Multimodal imaging may provide complementary information, but it does not automatically resolve uncertainty in response assessment. Its findings become interpretable only when the role of each modality, assessment timing, response thresholds, and rules for resolving discordant results are prespecified in the study protocol. Otherwise, adding imaging modalities may generate more conflicting signals without improving diagnostic accuracy. A more realistic objective is not to identify a single criterion that replaces RECIST, but to define the biological construct measured by each imaging method and to prespecify assessment timing, decision thresholds, and procedures for adjudicating discordant findings. Without such standardization, multimodal imaging may increase the volume of information without increasing confidence in the resulting conclusions.

### 3.3 Endpoint mismatch can generate unsupported therapeutic claims

Efficacy in studies of bone metastases encompasses at least three distinct constructs: antitumor effects within bone lesions, overall disease control at the patient level, and skeletal clinical benefit. A major limitation of the current literature is not simply the absence of bone-specific endpoints, but the frequent use of an outcome at one level to support a therapeutic claim at another. Bone-lesion antitumor endpoints describe changes in the tumor within bone and include bone-specific response, time to bone progression, bone-only progression, and concordance between bone and nonbone lesion responses. These measures are most directly relevant to whether a treatment controls tumor growth within bone, but their interpretation depends on lesion definitions, imaging methods, and event-adjudication rules. Bone-only progression indicates only that disease within bone was the first to meet a prespecified progression criterion. It does not exclude the influence of baseline bone burden, imaging sensitivity, or assessment frequency, and it cannot by itself establish bone-specific immune resistance.

Patient-level tumor outcomes, including objective response rate, systemic PFS, and OS, remain central to evaluating the clinical value of systemic therapy. They cannot, however, identify which organ or lesion drives treatment failure. Shorter systemic PFS may result from progression at any metastatic site, while OS is also affected by skeletal complications, treatment tolerance, and opportunities for subsequent therapy. Differences in patient-level survival therefore cannot substitute for direct evidence of bone-lesion response. Skeletal clinical outcomes include skeletal-related events, pathological fractures, spinal cord compression, the need for bone surgery or radiotherapy, pain, mobility, and analgesic use (37–44, 58). These outcomes reflect the functional and quality-of-life burden of bone metastases but are not equivalent to changes in tumor burden within bone. A reduction in fractures or a delay in the need for radiotherapy demonstrates skeletal protection, but it does not independently establish enhanced antitumor activity of ICIs. Conversely, radiographic response within bone may not translate directly into pain relief or functional improvement.

Studies should therefore avoid three common forms of endpoint substitution: using an association with OS to support immune sensitization within bone lesions, using a reduction in skeletal-related events to support an antitumor effect within bone, and using control of irradiated lesions to support systemic immune synergy. Bone-lesion antitumor outcomes, patient-level disease outcomes, and skeletal clinical benefits should be reported separately. Only then can investigators determine whether an intervention alters tumor activity within bone, the overall course of disease, or the clinical burden caused by bone metastases (**Figure 1**). The interpretive boundaries of commonly used bone-metastasis endpoints and assessment approaches are summarized in **Table 2**.

**Figure 1:**
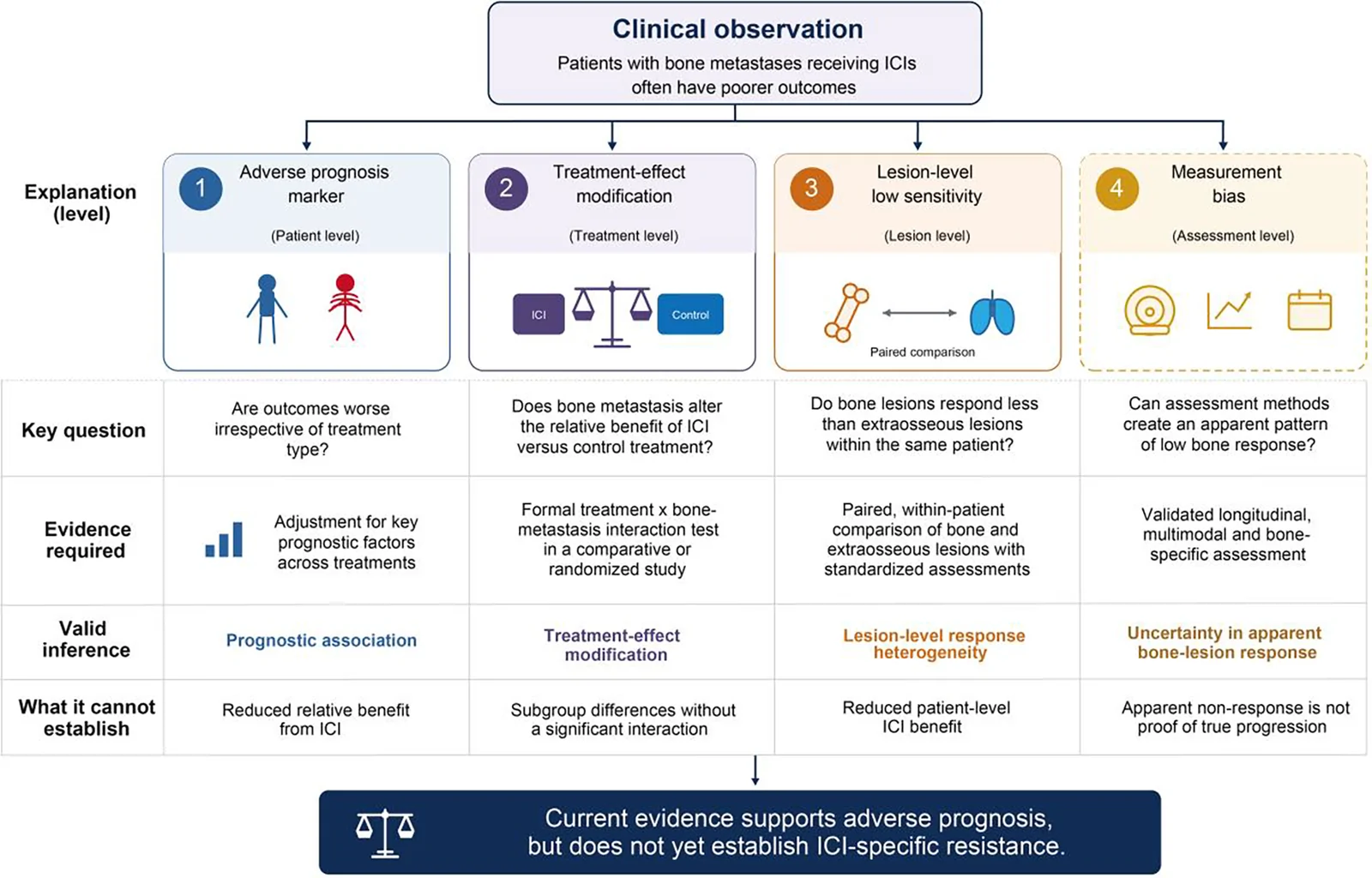
Framework for distinguishing adverse prognosis, treatment-effect modification, lesion-level response heterogeneity, and measurement bias in ICI-treated patients with bone metastases.

**Table 2:** Bone-metastasis endpoints, assessment methods, and the therapeutic claims they can support.

Endpoint/assessment	What it measures	Main limitation	Can support	Cannot establish
RECIST overall response	Overall measurable tumor burden	Most bone-only lesions are nonmeasurable	Systemic tumor response	Bone-specific response
MDA bone response	Structural change and remineralization in bone	Sclerosis is influenced by repair, radiotherapy, and bone-targeted therapy	Structural bone response	Validated ICI-specific bone response
Prespecified bone-lesion response	Lesion-level change according to predefined response criteria	Structural changes may reflect reparative remodeling rather than tumor regression	Bone-lesion response under the specified assessment framework	Systemic survival benefit
Bone-first progression	First site of progression	Influenced by baseline bone burden and assessment frequency	Pattern of progression	Bone-specific immune resistance
OS/systemic PFS	Patient-level clinical outcome	Confounded by disease burden, SREs, and subsequent therapy	Prognosis/overall disease control	Reversal of the local osteoimmune niche
SREs/pain	Skeletal morbidity	Not equivalent to tumor burden	Skeletal protection	ICI immune sensitization
Irradiated-lesion control	Local effect at treated lesions	Cannot separate direct radiotherapy effect	Local tumor control	Systemic abscopal immune effect

## 4 Mechanistic hypotheses: can the osteoimmune niche explain apparent bone-specific low sensitivity to ICIs?

Bone metastases can establish a local ecosystem that differs from both the primary tumor and other metastatic sites, but organ specificity does not in itself imply resistance to immune checkpoint inhibitors (ICIs). In this context, the osteoimmune niche is best viewed as a bidirectionally coupled system in which immune and stromal signals regulate osteoclast and osteoblast activity, while altered bone remodeling in turn reshapes the local cellular and signaling environment. For a mechanism within the osteoimmune niche to explain reduced responsiveness of bone lesions, at least three links would need to be established: the relevant state should be enriched in poorly responding bone lesions; changes in that state should correlate with bone-lesion response during ICI treatment; and targeting the state should produce benefits beyond general tumor reduction or skeletal protection. Few studies currently satisfy all three conditions.

The most biologically coherent candidate processes include impaired T-cell infiltration and effector function, together with interactions between myeloid immunosuppression and abnormal bone remodeling. Most supporting evidence, however, comes from breast or prostate cancer models of bone metastasis, pretreatment human tissue, or studies of nonbone tumors. Longitudinal studies directly comparing human bone lesions before and after ICI treatment, responding and progressing lesions, or paired bone and nonbone metastases remain limited. This section therefore presents working models that may explain the clinical observations rather than a unified and established pathway of resistance.

### 4.1 Early colonization provides biological context but does not explain ICI response

Normal bone marrow supports bone remodeling, hematopoiesis, and immune function. Interactions among osteoclasts, osteoblasts, stromal cells, vascular endothelial cells, and hematopoietic cells create a survival environment that disseminated tumor cells can exploit (59–71). CXCL12–CXCR4 signaling, E-selectin, and other adhesion mechanisms promote tumor-cell homing to and retention within the bone marrow (60, 72–75). Systemic factors released by the primary tumor may also alter marrow stromal and myeloid-cell states before metastases become radiographically detectable (60, 61, 73, 76). These studies primarily explain how tumor cells enter bone and establish early lesions. They do not show that established bone metastases are relatively insensitive to ICIs. Pathways involved in early homing may remain active in mature lesions, but their function may shift from colonization and retention toward regulation of immune-cell distribution, stromal architecture, or tumor-cell survival. Without direct functional evidence in established lesions, the involvement of CXCL12–CXCR4 in bone marrow homing does not justify the assumption that blockade of this pathway will restore ICI responsiveness.

As lesions expand, increased osteoclast activity or abnormal bone formation can release growth factors from the bone matrix and alter the vascular, stromal, and myeloid composition of the local environment, contributing to the classic vicious cycle of bone metastasis (59, 77–82). This process may provide a structural basis for immunosuppression. Its relevance to ICI response, however, depends on whether it further restricts antigen presentation, effector-cell infiltration, or local cytotoxic function, rather than on the presence of the vicious cycle itself.

### 4.2 Evidence for T-cell exclusion and dysfunction is suggestive but indirect

PD-1/PD-L1 blockade can produce local tumor control only if tumor-specific T cells can enter the lesion and maintain effector function. Abnormal vasculature, stromal deposition, and bone remodeling may interfere with these processes, but most available evidence remains indirect. Studies of prostate cancer bone metastases have linked abnormal osteogenesis with endothelial reprogramming and stromal changes (83–86). These alterations could plausibly affect perfusion, vascular permeability, and immune-cell migration, but low T-cell density in tissue sections does not by itself demonstrate impaired entry. Reduced T-cell abundance may also result from inadequate antigen presentation, local cell death, sampling location, or differences in treatment exposure. Direct observation of T-cell transit from the vasculature into tumor nests in human bone metastases remains lacking.

CXCL12–CXCR4 signaling and TGF-β are candidate pathways linking stromal changes to the spatial distribution of T cells. The role of CXCL12–CXCR4 in early bone marrow homing is well established, but whether this axis directly causes CD8^+^ T-cell exclusion in established bone lesions remains uncertain (60, 72–74). TGF-β released during bone resorption may contribute to fibrosis and immunosuppression. Models of nonbone tumors also suggest that TGF-β can restrict T-cell entry by reducing CXCR3-dependent migration (87–89). The magnitude of this effect in established human bone metastases, and whether it determines ICI response, have not been defined in longitudinal studies.

Paired-tissue analyses have shown that some bone metastases exhibit lower immune activity and reduced T-cell infiltration than the corresponding primary tumors while retaining PD-1 or PD-L1 expression (90). This indicates that bone lesions do not necessarily lack the molecular targets of ICIs. Target expression alone, however, does not establish PD-1/PD-L1 signaling as the dominant suppressive mechanism or show that T-cell function can be restored by checkpoint blockade. Similarly, coexpression of multiple inhibitory receptors may reflect functional exhaustion, but it may also accompany persistent immune activation and requires functional and treatment-response data for interpretation. Current findings are more consistent with the presence of T-cell paucity, spatial exclusion, or impaired function in some bone lesions than with a single dominant mechanism of ICI failure. Distinguishing among these states will require analyses that integrate spatial localization, effector function, and lesion-level response.

### 4.3 Myeloid–bone remodeling coupling is phenotype- and cancer-dependent

Myeloid cells may provide an important link between immunosuppression and bone remodeling within the metastatic niche. Osteoclasts arise from the myeloid lineage, and their differentiation is regulated in part through the RANK–RANKL–osteoprotegerin (OPG) axis, linking immune and stromal signaling to skeletal remodeling (23, 91–93). Conversely, changes in osteoclast activity and bone resorption can alter the local availability of matrix-derived mediators and thereby influence stromal, myeloid, and lymphocyte states (23, 91, 94). Myeloid-derived suppressor cells, macrophages, osteoclast precursors, and related populations may therefore contribute simultaneously to immune regulation and bone remodeling rather than representing independent components of the niche (23, 91, 94–96). The categories “myeloid-derived suppressor cells,” “tumor-associated macrophages,” and “osteoclast precursors,” however, are neither stable nor fully distinct. Studies differ in the markers, models, and functional criteria used to define these populations, and some states may lie along a lineage continuum. An increase in myeloid-cell abundance therefore does not establish that these cells drive reduced ICI responsiveness in bone lesions without spatial and functional validation.

In osteolytic and mixed lesions, increased bone resorption can release matrix-derived factors such as TGF-β, providing one mechanism through which skeletal remodeling may feed back on tumor growth and local immune regulation (88, 92, 93). This osteoclast–matrix factor–immunosuppression sequence is biologically coherent, but it is supported mainly by breast cancer studies and preclinical models. Whether it determines bone-lesion response during ICI treatment in humans remains unclear. Osteoblastic lesions arise in a different mechanistic context. Studies of prostate cancer emphasize Wnt-related abnormal bone formation, vascular reprogramming, and specific macrophage states (83, 84, 86, 97–99). These findings cannot be transferred without qualification to tumor types dominated by osteolytic disease. Bone metastases in NSCLC also show substantial phenotypic and molecular heterogeneity (19, 21), making it unlikely that a single RANKL-, TGF-β-, or Wnt-centered pathway explains all lesions. Abnormal bone remodeling and myeloid immune states may reinforce one another, but the composition and treatment dependence of this feedback are likely to vary by cancer type and lesion phenotype. A more clinically relevant question is which functional state recurs within a defined bone phenotype and represents a tractable therapeutic vulnerability.

### 4.4 Cancer type and treatment exposure preclude a unified niche model

Bone metastases encountered in clinical practice have often been exposed to systemic therapy, radiotherapy, or bone-targeted treatment. Their local environment is therefore not an untreated natural state but the product of tumor evolution and prior therapeutic exposure. Different treatments may simultaneously alter tumor burden, bone remodeling, and immune composition, yet most cross-sectional tissue studies cannot determine whether an observed niche feature was present before treatment or was selected, induced, or amplified during therapy.

When bone-dominant progression develops after treatment, at least three competing explanations should be considered: pre-existing local immune restriction; a suppressive state selected or induced during therapy; and nonimmune escape related to tumor clones, local drug exposure, prior radiotherapy, or lesion architecture. Without paired pretreatment and post-treatment sampling and lesion-level comparisons, these processes cannot be distinguished. The immune phenotype of a progressing lesion should not be used retrospectively to infer the original mechanism of resistance. Differences among cancer types further limit the development of a single niche model. Prostate cancer bone metastases are more strongly associated with abnormal osteogenesis and vascular–stromal reprogramming, whereas breast cancer more often involves enhanced osteoclast activity and TGF-β-related osteolytic cycles. NSCLC shows greater heterogeneity in bone phenotype and molecular features (19, 82–84, 89, 90, 93, 97, 100, 101). Broad processes such as abnormal bone remodeling, myeloid enrichment, and impaired T-cell function may be shared across cancers, but uniform cellular states or a single therapeutic target are less likely. The osteoimmune niche is therefore better understood as a set of dynamic local states shaped jointly by cancer type, bone-remodeling phenotype, and treatment exposure, rather than as a fixed and universal program of ICI resistance.

## 5 Therapeutic targeting of the osteoimmune niche: skeletal benefit, local control, or immune sensitization?

The field of bone metastasis treatment does not lack biologically plausible combination strategies. What remains limited is evidence that can distinguish the source of therapeutic benefit. Accordingly, this section evaluates whether candidate interventions have demonstrated skeletal protection, local antitumor control, or evidence sufficient to support ICI sensitization.

Among currently available approaches, RANKL inhibition and bone-directed radiotherapy have established clinical roles based on skeletal protection and local control, respectively. Chemotherapy and antiangiogenic therapy may indirectly alter the environment of bone lesions, but their effects cannot be assigned specifically to the bone niche. TGF-β signaling and myeloid–bone remodeling interactions have relatively coherent mechanistic support, whereas evidence for CXCL12–CXCR4 is derived largely from studies of early homing and colonization. Bone-targeted delivery, engineered immune or hematopoietic cells, and multiple-checkpoint blockade remain largely at the proof-of-concept stage.

### 5.1 RANKL inhibition: target engagement without established evidence of immune sensitization

Denosumab inhibits osteoclast maturation and bone resorption by blocking RANKL. Its ability to reduce or delay skeletal-related events is supported by randomized trials, meta-analyses, and clinical guidelines (38, 102, 103). Because the RANK–RANKL axis contributes to both bone remodeling and immune regulation, combining denosumab with ICIs has a plausible biological rationale (88, 92, 93, 104, 105). Suppression of bone turnover, however, establishes target engagement but does not show that immune suppression within bone has been relieved. Case series and retrospective studies in NSCLC and melanoma have associated the combination of ICIs and denosumab with improved bone-lesion control or survival (106–109). These findings provide a clinical signal that warrants further study, but they do not reliably distinguish immune synergy from patient selection. A multicancer retrospective study of 268 patients found no significant overall improvement in OS or PFS and reported only exploratory differences in a small subgroup defined by treatment sequence (110). The inconsistency of these findings may reflect not only differences in cancer type and ICI regimen, but also variation in how observational studies define denosumab exposure. Such analyses are particularly vulnerable to confounding by indication and time-related bias. Patients with a greater bone burden, more severe pain, or a higher risk of skeletal-related events are more likely to receive denosumab. Conversely, patients with better disease control and longer survival have more opportunity to accumulate prolonged exposure to the combination. Grouping patients by duration of combined treatment may introduce guarantee-time bias. Failure to distinguish denosumab initiated before ICIs, started concurrently, or added after treatment onset may also combine biologically and clinically distinct settings. Concomitant radiotherapy, chemotherapy, bisphosphonates, and subsequent treatments may further influence observed outcomes.

Compared with zoledronic acid, denosumab is more effective in delaying some skeletal-related events, but it has not shown a clear overall survival benefit (102). Even if differences between the two bone-targeted agents are observed, clinical outcomes alone cannot establish an immune effect specific to RANKL blockade. Evidence of immune sensitization would require antitumor activity within bone beyond skeletal protection, accompanied by prespecified changes in osteoclast activity, myeloid states, or T-cell function. The skeletal protective value of denosumab is established, whereas its ability to sensitize tumors to ICIs remains a clinical signal rather than a proven effect. The next step should not be another unadjusted comparison of survival between patients who did and did not receive the combination. Studies should instead determine whether denosumab improves bone-lesion control beyond reducing skeletal-related events and whether this benefit is accompanied by corresponding local pharmacodynamic changes.

### 5.2 Bone-directed radiotherapy: local control without established immune sensitization

Bone-directed radiotherapy relieves pain, controls local tumor growth, and reduces the risk of structural complications. Its clinical value does not depend on an immune-synergy hypothesis. Radiotherapy may also promote antigen release, local inflammation, and immunogenic cell death, but these effects are not uniformly stimulatory. Irradiation can damage intratumoral and circulating lymphocytes and may suppress hematopoietic and immune function when active bone marrow is exposed (111–120). Control of an irradiated lesion should therefore be interpreted first as a direct local effect of radiotherapy rather than as evidence of ICI sensitization. Studies of bone radiotherapy combined with ICIs have consisted mainly of case reports and retrospective cohorts. Improved survival has been reported in patients with melanoma receiving the combination, and regression of nonirradiated lesions has been described in individual cases of bone metastasis and NSCLC (121–123). These observations establish that abscopal responses can occur, but they do not define their frequency, reproducibility, or incremental effect relative to ICI treatment alone. Recent observational studies and meta-analyses in NSCLC have also reported possible benefits in survival, nonbone lesion response, or skeletal-related events, but the included studies were heterogeneous in treatment selection, disease burden, and timing of radiotherapy.

Different clinical settings for bone radiotherapy should not be treated as a single exposure. Initial treatment of oligometastatic disease aims at disease ablation. Radiotherapy for bone oligoprogression during ICI treatment seeks to prolong control with the ongoing systemic regimen. In patients with widespread symptomatic bone metastases, the primary goals are pain relief and preservation of structural stability. Differences in PFS or OS observed across these settings therefore have distinct clinical meanings. Evaluation of systemic immune sensitization also requires separate assessment of irradiated and nonirradiated lesions, together with consideration of dose, fractionation, treatment volume, marrow exposure, and the sequence of radiotherapy relative to ICIs. Response of irradiated lesions alone cannot distinguish direct radiotherapy effects from immune synergy. Responses in nonirradiated lesions are also difficult to interpret without an appropriate comparator because delayed effects of ICIs and patient selection remain alternative explanations. The local therapeutic value of bone radiotherapy is established, but whether it consistently enhances ICI responses in nonirradiated bone or nonbone lesions has not been demonstrated. Isolated abscopal responses and observational survival associations are insufficient to conclude that the osteoimmune niche has undergone systemic immune remodeling.

### 5.3 Systemic combinations: clinical signals without bone-specific attribution

Some studies suggest that the adverse association of bone metastases with outcomes may be weaker among patients receiving chemotherapy plus ICIs or antiangiogenic therapy plus ICIs than among those receiving ICI monotherapy (5, 46–48). These findings have sometimes been interpreted as evidence that combination treatment partly overcomes local immune suppression within bone, but current data do not support such a specific attribution. Chemotherapy can reduce systemic tumor burden and alter antigen release and myeloid-cell composition, while also causing lymphopenia and bone marrow suppression. Antiangiogenic therapy may improve abnormal vasculature and tissue perfusion, but it also has independent systemic antitumor activity. Improvement in bone lesions during combination treatment may therefore reflect greater overall treatment efficacy rather than selective alteration of the osteoimmune niche. More importantly, existing studies generally lack paired response assessment of bone and nonbone lesions, bone tissue collected before and after treatment, and longitudinal evaluation of local vascular, myeloid, and T-cell states. Chemotherapy and antiangiogenic therapy therefore illustrate why clinical improvement should not automatically be attributed to niche reversal. They should not yet be regarded as validated bone microenvironment-directed treatments.

### 5.4 Mechanism-directed candidates: unequal biological and translational support

The evidence supporting different mechanistic targets varies substantially. TGF-β links bone resorption, stromal remodeling, fibrosis, and immunosuppression and is among the more biologically coherent candidate pathways (87, 88). Local TGF-β inhibition has improved immune states and antitumor activity in models of bone metastasis (124), but no evidence shows that this approach improves ICI efficacy in human bone metastases. Because TGF-β has broad roles in normal tissue homeostasis and repair, systemic inhibition may be constrained by toxicity and a narrow therapeutic window. Local delivery, in turn, presents challenges related to manufacturing, distribution, and long-term safety. CXCL12–CXCR4 signaling has a well-established role in bone marrow homing, retention, and early colonization (60, 72, 74, 125), but its connection to reduced ICI responsiveness in established macroscopic bone lesions is less direct. CXCR4 blockade may be more relevant to early colonization, minimal residual disease, or prevention of bone metastasis. Its use in established bone metastases would require evidence that the pathway continues to regulate immune-cell spatial distribution or tumor-cell survival in progressing lesions. Because CXCL12–CXCR4 also supports normal hematopoietic stem-cell homeostasis, systemic intervention may produce substantial effects unrelated to the tumor.

The appeal of myeloid-directed therapy lies in the dual roles of myeloid-derived suppressor cells, macrophages, and osteoclast precursors in immunosuppression and bone remodeling (83, 94–96). Myeloid suppression, however, is not a single or stable therapeutic target. These cell states overlap in marker expression and lineage and vary according to cancer type, bone-remodeling phenotype, and treatment exposure. Broad depletion of myeloid cells may also impair normal bone remodeling, antimicrobial defense, and tissue repair. The main obstacle is therefore not a lack of candidate drugs, but the absence of a reproducible pathogenic cell state with a stable functional definition, bone-lesion specificity, and treatment dependence. Bone-targeted drug delivery, engineered immune or hematopoietic cells, and multiple-checkpoint blockade remain largely at the proof-of-concept stage (124, 126–129). These platforms may improve local drug exposure or antigen recognition, but bone targeting is not equivalent to bone-metastasis specificity. Enhanced immune recognition also does not necessarily overcome vascular, stromal, myeloid, or metabolic constraints. Likewise, coexpression of multiple immune checkpoints does not establish that each pathway is therapeutically relevant. Until bone-lesion-specific functional and interventional evidence becomes available, these approaches are better regarded as future technological directions than as near-term clinical strategies.

Overall, no current intervention has simultaneously demonstrated antitumor benefit within bone lesions, modification of the local niche, and an additional ICI effect relative to an appropriate comparator. The established clinical value of RANKL inhibition and bone-directed radiotherapy rests on skeletal protection and local control, respectively. The effects of chemotherapy and antiangiogenic combinations cannot be attributed specifically to bone, whereas TGF-β inhibition, myeloid targeting, CXCL12–CXCR4 blockade, and other platforms remain primarily at the stage of mechanistic validation or preclinical development. Improved survival or local lesion control after combination treatment therefore cannot by itself establish therapeutic remodeling of the osteoimmune niche (**Figure 2**).

**Figure 2:**
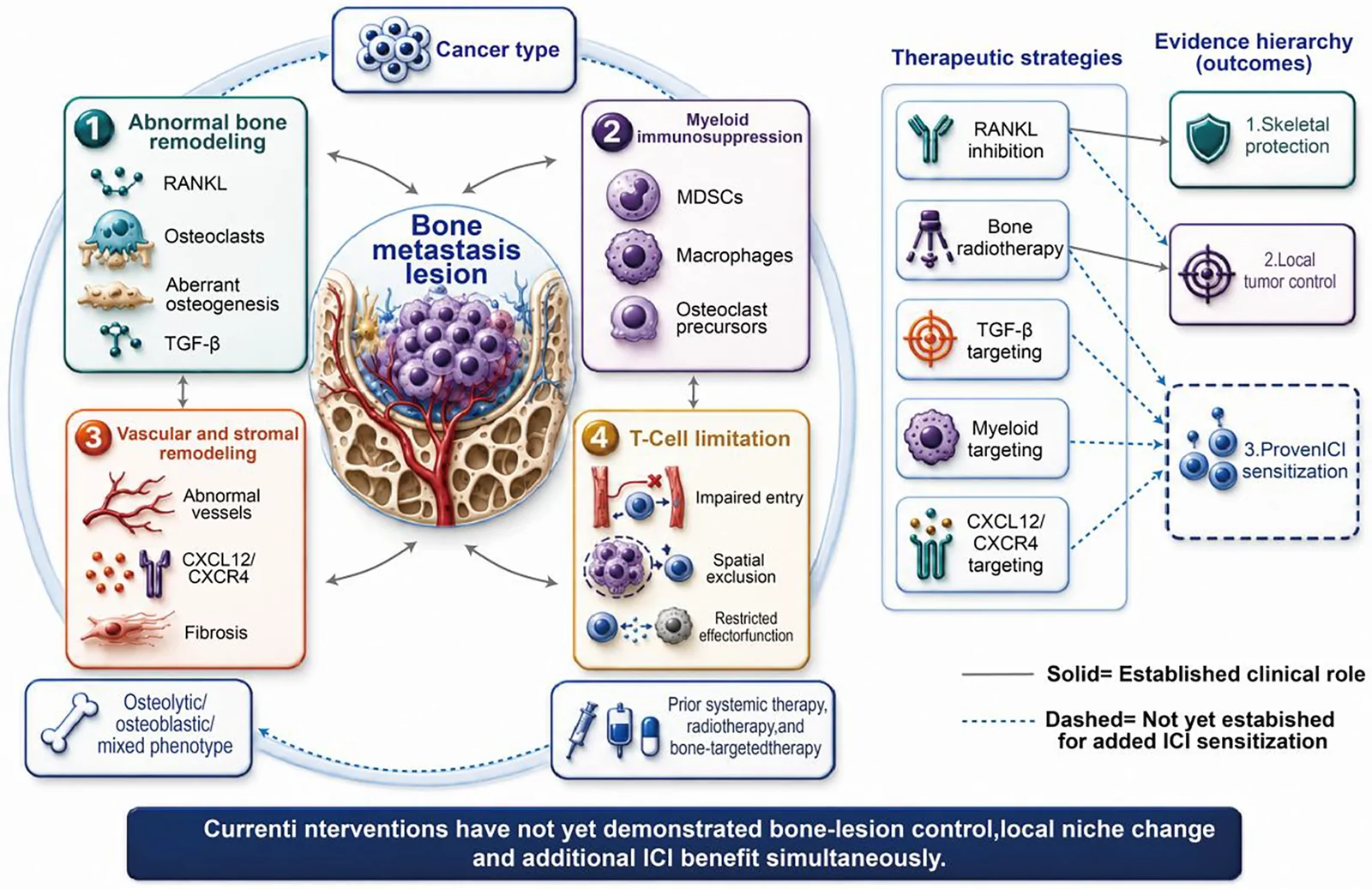
Osteoimmune niche states, candidate therapeutic strategies, and the hierarchy of clinically supported outcomes in bone metastases.

## 6 Stratifying bone metastases: what current markers can— and cannot—tell us

Classifying patients simply according to the presence or absence of bone metastases is insufficient to characterize disease severity or identify local niche states that may influence response to immune checkpoint inhibitors (ICIs). A solitary bone lesion, extensive involvement of the axial skeleton, and diffuse bone marrow infiltration differ substantially in tumor burden, hematopoietic impact, and treatment tolerance. Osteoblastic, osteolytic, and mixed lesions may also arise within distinct bone-remodeling and immune contexts. Even if bone metastases are associated with reduced local sensitivity to ICIs, this is therefore unlikely to reflect a single biological feature shared by all patients. Current stratification research faces a fundamental trade-off. Easily accessible markers generally lack bone-lesion specificity, whereas markers closest to local mechanisms are limited by sampling, technical constraints, and lesion representativeness. Available candidate variables can help characterize disease burden or generate hypotheses about the osteoimmune niche, but no marker has been shown to identify patients who derive additional benefit from a corresponding microenvironment-directed intervention. This section therefore does not seek to identify a single optimal biomarker. Instead, it examines what different types of information can currently establish and which therapeutic inferences remain beyond the available evidence.

### 6.1 Clinical variables control confounding rather than identify immune sensitivity

The number of bone lesions, overall skeletal burden, bone marrow involvement, skeletal-related events, and concurrent visceral metastases are among the most readily available stratification variables. Their primary value is not to predict the sensitivity of bone lesions to ICIs, but to describe disease severity and reduce the risk of mistaking differences in baseline prognosis for bone-specific treatment resistance. Most ICI studies continue to record only the presence or absence of bone metastases and rarely distinguish among limited skeletal disease, bone-dominant disease, extensive axial involvement, and diffuse bone marrow infiltration (1–5, 31, 45, 130). This binary classification combines patients with markedly different biological and clinical states. An asymptomatic solitary rib lesion does not carry the same disease burden, hematopoietic consequences, or implications for treatment tolerance as extensive involvement of the spine, pelvis, and bone marrow, yet these conditions are usually grouped within the same exposure category in retrospective studies.

Bone marrow involvement may represent a particularly important but inadequately measured dimension of disease. Replacement of normal marrow by tumor can cause anemia, thrombocytopenia, reduced lymphocyte reserve, and impaired tolerance of systemic therapy. These effects, however, should not be interpreted directly as evidence of immunosuppression within the bone marrow. Conventional CT and bone scintigraphy have limited sensitivity for early marrow infiltration, and definitions and assessment methods remain inconsistent. Bone marrow involvement is therefore better regarded as a disease-burden variable that requires standardized documentation than as an established marker of local immune function. Skeletal-related events and lesion location also primarily reflect clinical burden. Extensive involvement of the spine and pelvis may impair hematopoiesis, mobility, and performance status, whereas lesions in long bones are more likely to cause structural instability and pathological fractures. Spinal cord compression and the need for bone surgery or radiotherapy may interrupt systemic treatment and reduce opportunities for subsequent therapy (37–44, 58). These events may explain part of the observed survival disadvantage, but they cannot determine whether RANKL, TGF-β, or a particular myeloid state governs the response of bone lesions. Concurrent liver, brain, and other visceral metastases represent another important source of confounding. Patients with bone-only disease follow a different clinical course from those with multiorgan metastases (40–42). Failure to account for liver involvement, the number of metastatic organs, and overall tumor burden may therefore overestimate the adverse effect attributed to bone metastases (1, 31, 33–35, 131). The main role of clinical and anatomical variables is thus to define study populations, balance baseline risk, and support prognostic interpretation rather than directly select patients for microenvironment-directed treatment. These variables address how advanced a patient’s disease is, not which local mechanism is therapeutically actionable.

### 6.2 Biomarker accessibility comes at the cost of bone specificity

Current biomarkers can be broadly divided into three groups: circulating markers of systemic disease state, imaging and metabolic measures of bone remodeling, and tissue or spatial markers derived directly from local lesions. Each provides useful information, but they do not offer the same degree of mechanistic resolution. The neutrophil-to-lymphocyte ratio, platelet-to-lymphocyte ratio, systemic immune-inflammation index, and other inflammatory or nutritional markers are readily available, and some cohorts of patients with NSCLC and bone metastases have associated them with outcomes during ICI treatment (132, 133). These measures, however, are influenced by infection, corticosteroid use, pain, radiotherapy, liver metastases, and overall tumor burden. Even if they consistently identify patients with poorer prognosis, they cannot determine whether the source of risk lies within bone lesions. They are therefore better viewed as systemic prognostic markers than as biomarkers of the osteoimmune niche. Circulating tumor DNA also offers advantages for dynamic monitoring, but it primarily reflects overall tumor burden and clonal evolution. Unless combined with lesion-specific mutations or other localization information, changes in circulating tumor DNA generally cannot establish whether the signal originates from bone or nonbone disease. An association with overall treatment response does not demonstrate that the niche within bone has changed.

Bone-turnover markers and imaging features of bone remodeling are more closely related to bone-lesion biology than general circulating inflammatory markers, but they still lack tumor specificity. Age, renal function, fractures, endocrine therapy, radiotherapy, and bone-targeted agents can all affect bone-turnover measurements (134, 135). A single elevated value cannot reliably distinguish active tumor, reparative bone formation, and treatment effects. Importantly, in the study by Ibrahim et al., biomarker associations with bone response were defined using both the MDA criteria and PERCIST, illustrating that the apparent performance of a circulating biomarker also depends on the response-assessment framework used to define the outcome (134). This highlights the dependence of biomarker interpretation on the response-assessment framework discussed in Section 3. Osteoblastic, osteolytic, and mixed lesions may provide a broad form of mechanistic stratification because they may reflect different states of bone-remodeling–immune coupling (82–84, 88, 89, 93). Imaging phenotype alone, however, cannot identify which pathway is treatment-dependent or directly guide selection of RANKL-, TGF-β-, or Wnt-directed interventions.

Local tissue, single-cell, and spatial analyses provide the closest view of the osteoimmune niche, but they also have the greatest limitations in representativeness. Available bone specimens are usually obtained from symptomatic lesions, lesions requiring surgery, or lesions with diagnostic uncertainty. Such samples may disproportionately represent structurally unstable disease, treatment-resistant lesions, or specific anatomical sites. A single sampled lesion may not represent other bone lesions within the same patient or the broader population of patients with bone metastases. Decalcification, fixation, and tissue processing may also compromise RNA quality, protein antigens, and spatial architecture (84, 90, 99). Human studies have identified immune, stromal, and vascular differences between bone metastases and primary tumors and across bone-remodeling phenotypes. These findings first establish heterogeneity within bone lesions; they do not by themselves show that a particular cell state drives reduced sensitivity to ICIs. Myeloid enrichment or T-cell exhaustion observed in progressing lesions may be a cause of treatment failure, but it may also result from persistent tumor growth and treatment pressure. Without paired pretreatment and post-treatment sampling and functional validation, cross-sectional differences cannot establish causal direction. Similarly, PD-1/TIGIT coexpression, SPP1-associated macrophages, and other single-cell subgroup labels should not be treated as patient-selection biomarkers solely because they are enriched in a particular dataset. Single-cell clustering and annotation are influenced by sample processing, analytical methods, and reference frameworks, and the same label may not represent the same functional state across studies. Functional states such as spatial T-cell exclusion, myeloid-dominant suppression, high osteoclast activity, or abnormal osteogenic–vascular reprogramming may provide more useful hypotheses for validation than isolated cell-subpopulation labels.

Biomarker requirements should be defined according to their intended use rather than treated as universal criteria. Prognostic biomarkers may support risk stratification without requiring bone-lesion specificity or mechanistic validation, whereas predictive biomarkers require evidence that the marker identifies differential treatment benefit, ideally through prespecified treatment-by-biomarker interaction analyses. Lesion-monitoring biomarkers require longitudinal association with bone-lesion status, while pharmacodynamic biomarkers should demonstrate treatment-induced changes in the targeted biological pathway. Accessibility, bone-lesion specificity, longitudinal measurability, and mechanistic actionability should therefore be regarded as purpose-dependent attributes rather than requirements that every useful biomarker must simultaneously satisfy.

## 7 What evidence would change the field? From association to causal inference

The association between bone metastases and poorer outcomes after ICI treatment has been observed repeatedly, but further replication of this association alone is unlikely to change clinical understanding. The priority is now to resolve three related but distinct questions: whether bone metastases modify the relative efficacy of ICIs, whether bone lesions are less responsive than nonbone lesions under standardized assessment, and whether candidate osteoimmune states are causally linked to treatment response and therapeutically reversible. These questions require different comparators, endpoints, and temporal designs. Larger cohorts or higher-dimensional biological data cannot compensate for a mismatch between the therapeutic claim and the study design used to test it.

### 7.1 Evidentiary designs for the key unresolved questions

Determining whether bone metastases modify ICI efficacy requires comparison with an appropriate control treatment, with bone-metastasis status or the candidate biomarker prespecified and its interaction with treatment tested directly. Determining whether bone lesions are relatively less sensitive should instead prioritize prospective within-patient comparisons of bone and nonbone lesions under standardized assessment. Lesion phenotype, measurable soft-tissue components, prior local treatment, and imaging methods should be documented prospectively so that apparent site-specific differences can be distinguished from lesion selection and measurement effects.

Studies of microenvironment-directed interventions require an additional level of evidence. Bone-lesion antitumor endpoints should be defined prospectively and linked to longitudinal local pharmacodynamic measurements of the biological state targeted by the intervention. For radiotherapy studies, irradiated and nonirradiated lesions should be reported separately. Protocols should also prespecify how purely osteoblastic lesions, reparative sclerosis, newly detected sclerotic lesions, discordant responses, and previously irradiated lesions will be handled. When nonbone progression or death occurs before bone progression can be confirmed, competing-risk methods or other appropriate approaches should be considered. A biological change without corresponding bone-lesion control may indicate target engagement or an accompanying treatment effect, but it would not by itself establish that the altered pathway mediates therapeutic benefit.

### 7.2 A pragmatic and falsifiable research agenda

Progress is more likely to come from a sequence of linked studies than from expecting a single trial to establish treatment interaction, lesion-level response, and mechanism simultaneously. First, randomized trial datasets and prospective cohorts should establish whether bone metastases reproducibly modify the relative treatment effect of ICIs. Second, prospective lesion-level studies should determine whether reduced responsiveness of bone lesions can be reproduced under standardized imaging and lesion-selection rules. Third, mechanism-focused window-of-opportunity or pharmacodynamic studies should test whether prespecified osteoimmune states change after intervention and whether those changes track with bone-lesion response. Longitudinal tissue sampling would provide the strongest local mechanistic evidence, but repeated bone biopsies are likely to be feasible mainly when lesions are safely accessible or tissue acquisition is clinically indicated. When serial biopsy is impractical, multimodal imaging, circulating measurements paired with lesion-level imaging, clinically indicated surgical specimens, and carefully designed window-of-opportunity studies can provide complementary longitudinal evidence. These approaches can help characterize temporal changes in bone lesions while reducing reliance on repeated invasive sampling. Biomarker-enriched trials should follow only after a candidate niche state can be measured reproducibly and linked to both lesion-level outcome and a biologically matched intervention.

Each stage should allow the central hypothesis to be rejected. If prespecified interaction testing does not demonstrate treatment-effect heterogeneity, bone metastases should not be considered an ICI-specific predictive factor. If standardized lesion-level studies do not reproduce reduced responsiveness within bone, models of bone-specific low sensitivity should be reconsidered. If intervention-induced biological changes are not associated with bone-lesion response, the corresponding pathway should not be regarded as a mediator of therapeutic benefit. Only after withstanding these tests can candidate osteoimmune states progress from biologically plausible explanations to clinically meaningful targets for patient stratification and treatment selection.

## 8 Conclusions

Current evidence consistently associates bone metastases with poorer outcomes after ICI treatment, but it does not establish that bone metastases independently reduce the relative benefit of ICIs. Discordant responses between bone and nonbone lesions suggest that bone may represent a site of relative low sensitivity in some patients, but direct longitudinal evidence for an organ-specific immune mechanism in humans remains limited. The immune-sensitizing effects of denosumab, bone-directed radiotherapy, and other microenvironment-directed strategies have not been reliably demonstrated. Future studies should focus on candidate osteoimmune niche states and use bone-lesion-specific endpoints, longitudinal pharmacodynamic evidence, and formal treatment-interaction testing to determine whether these states have genuine value for patient stratification and treatment selection.
